# Mutation of the histidin residue of the DRH motif in vasopressin V2 receptor expression and function

**Published:** 2010

**Authors:** H. Mir Mohammad Sadeghi, M. Rabbani, A. Jafarian, H. Najafzadeh, L. Safaeian

**Affiliations:** 1 Department of Pharmacology and Toxicology; 2 Isfahan Pharmaceutical Sciences Research Center, School of Pharmacy and Pharmaceutical Sciences, Isfahan University of Medical Sciences, Isfahan; 3 Department of Pharmacology and Toxicology, School of Veterinary Medicine, Shahid Chamran University, Ahvaz, Iran

**Keywords:** V2 receptor, Nested PCR, COS-7 cells, cAMP

## Abstract

**Background and the purpose of the study:**

Vasopressin type 2 receptor (V2R), a G protein coupled receptor (GPCR), plays an important role in the regulation of renal antidiuretic function. The highly conserved DRH motif is essential for G protein signaling of V2R; however its role especially regarding the histidin residue is not fully understood..

**Methods:**

Site directed mutagenesis was performed with replacements of the histidin to isoleucine by using nested polymerase chain reaction. ELISA was performed for receptor expression assay and the adenylyl cyclase activity assay was performed for functional characterization of DRI mutation on V2R signaling.

**Results and major conclusion:**

The adenylyl cyclase activity assay in COS-7 cells showed no difference in the amount of cAMP production between the wild type and the mutant V2 receptors. The V2 receptor expression was not changed in the presence of this mutation using ELISA assay. These results suggest that the role of histidin residue is not critical in the V2 receptor function, however further mutagenesis studies are required to define the role of this motif in V2R function.

## INTRODUCTION

Vasopressin type 2 receptor (V2R) is a rhodopsinlike G protein coupled receptor (GPCR) that plays an important role in water homeostasis in physiological and pathological conditions (1). It is positively linked to the Gs protein and its second messenger is cAMP (2). A number of common receptor regions are involved in the signal transduction pathways of GPCRs (3). One of the highly conserved motifs is the triplet of amino acids; glutamic acid/aspartic acid- arginine-tyrosine (DRY motif) which is located at the boundary between the third transmembrane region and the second intracellular loop (4). This segment of GPCRs has been shown to play an important role in cell surface delivery, internalization, ligand binding affinities, phosphorylation, desensitization and normal receptor function (5, 6). By forming intramolecular interactions, DRY motif has a pivotal role in the regulation of GPCR conformations in signaling and intracellular trafficking (4). Some mutations in this motif result in receptors with impaired signal transduction which are responsible for certain human diseases (7).

Congenital nephrogenic diabetes insipidus (NDI) is a rare disease with impairment of water reabsorption in renal collecting ducts and mutations in the V2R (8, 9). In V2R, the DRY motif is a DRH and one of the first-characterized mutations in NDI was R137H in this motif with inability of ligand-bound receptor to activate G proteins (9, 10). Despite extensive studies in other receptors, there are only limited findings about mutations in aspartate and arginine residues in V2R (11–13). Since there is no data available on the role of histidin residue in DRH motif in V2R, it was decided to use site directed mutagenesis in this region as DRI to gain some new insights into the receptor-function relationship.

## MATERIAL AND METHODS

### 

#### Plasmids and constructs

cDNA of the wild type V2R in pcDNA3 vector obtained from Dr. Birnbaumer's laboratory (8) was used as the template for the construction of the mutant V2R. A pair of primers was designed for DRI (aspartate-arginine-isoleucine) mutant by using WDNASIS26 program (Hitachi Software Engineering Co., Tokyo, Japan). Two pairs of outer and inner sense and antisense primers in cDNA of V2R and pcDNA3 vector were also designed.

**Figure 1:**
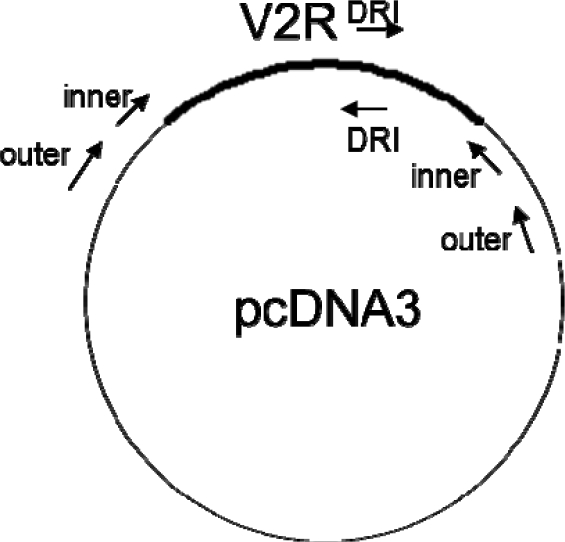
Schematic presentation of pcDNA3 plasmid containing wild type V2R sequence (shown in bold). Primers used for the PCR amplification of this sequence and creation of DRI mutation are also disclosed. Three pair of primers (outer, inner and DRI) were utilized in this study.

A schematic presentation of pcDNA3 plasmid containing wild type V2R and the location of the above mentioned primers is shown in figure 1.

The primer sequences were as follows: sense (5’-cgctggaccggatccgtgccatc-3’) and anti-sense (5’-gatggcacggatccggtccagcg-3’) for DRI mutant, sense (5’-cccagcaacagcagccag-3’) and anti-sense (5’-tcatcctcacagtcttgg-3’) for inner in V2R, sense (5’-ccaccacttccgctgtgc-3’) and anti-sense (5’-acccaacagctcctcacg-3’) for outer in V2R, sense (5’-gtggatagcggtttgact-3’) and anti-sense (5’-ggtcaaggaaggcacgg-3’) for inner in pcDNA3 and sense (5’-tgggtggactatttacgg-3’) and anti-sense (5’-gttctttccgcctcaga-3’) for outer in pcDNA3.

Nested polymerase chain reaction (PCR) was used for the synthesis of the mutant V2 receptor insert. The first PCR was performed using 50 ng cDNA of V2R (as template), 5 µl 10X PCR buffer containing 2 mM MgCl2 (Biotools, B & M Labs, Madrid, Spane), 0.5 mM dNTP (Eurobio Laboratories, France), 5 U Taq DNA polymerase (Biotools), 2.5 µM of each sense and anti-sense of DRI with outer primers in V2R in 2 steps (total volume of 50 µl). The PCR conditions were set as follows: 1 cycle (94°C for 5 min), 33 cycles (94oC for 1 min, 55oC for 2 min, 72°C for 2 min), and 1 cycle (72°C for 20 min) in a Thermostable DNA Termocycler (Bio-Rad Laboratories, California, USA). After electrophoresis on 0.7% agarose gel (Roche, Germany) and determination of the size of the obtained DNA, the products of the first PCR were used as the template for the second (nested) PCR with sense and anti-sense of inner primers in V2R (14).

The PCR products were confirmed by staining of the agarose gel with ethidium bromide using a UV transilluminator, and the obtained DNA bands were detected. The products containing the desired mutation were digested by BamHI, EcoRI and XbaI restriction enzymes (Fermentas). DNA was extracted from the agarose gel using QIAquic kit (Qiagen, Valencia, CA, USA). The DRI insert was ligated to pcDNA3 vector with a molar ratio of 3/1 (insert to vector). Transformation was performed using *E.coli* HB101 competent cells (Cinnagen, Tehran, Iran) with heat shock method and plasmid were prepared by alkaline lysis procedure using plasmid preparation kit from Bio-Rad, USA (15, 16).

#### Cell culture and transfection

COS-7 cells (Pasteur Institute, Tehran, Iran) were grown in DMEM (Dulbecco's modified eagle medium), with 10% FBS (fetal bovine serum) (Gibco, BRL Life Technologies, Glasgow, Scotland), penicillin (50 U/ml), and streptomycin (50 µg/ml) (Sigma, St. Louis, USA) in an incubator (5% CO2) at 37°C. For transient expression, cells were placed at a density of 5×105 cells per well in a 24-well plate and transfection was performed using modified Luthman and Magnusson method (8). Briefly, each plate was treated with 800 µl of Hanks buffer, pH of 7 (Sigma), containing 3 µg plasmid DNA of mutant or wild type V2R (0.5-2 µg) mixed with 0.5 mg/ml diethylaminoethyl-dextran (Sigma). The cells were incubated for 20 min at room temperature and then treated with 100 µM Chloroquine (Sigma) in DMEM containing 2% FBS. After 3 hrs incubation at 37°C, the cells were exposed to 10% dimethyl sulfoxide (Sigma) in Hanks buffer for 2 min, rinsed with DMEM, and treated with normal growth medium at 37°C.

#### ELISA

COS-7 cells were transiently transfected with the wild type or mutant V2R (the receptor containing the HA epitope at the C or N terminus) (11). For receptor expression assay, ELISA was performed using the 12CA5 monoclonal antibody (Sigma) against N-terminal epitope of V2R (8). Briefly, following 24 hrs transfection, cells were placed at a density of 3-5×105 cells per well in a 96-well plate. Then the medium was removed and 1 µg of 12CA5 of antibody/100 µl solution was added. After one hour incubation at 37°C, the cells were fixed with 4% formaldehyde in PBS. Wells were incubated with a 1:2500 dilution of horseradish peroxidase-conjugated anti-mouse IgG in PBS (Sigma) at 37°C for 2 hrs. The enzymatic reaction was induced by H2O2 and O-phenylenediamine (2.5mM) (Sigma) and then the reaction was stopped by the addition of 3M HCl. The optical density was measured at 492 nm in a microplate reader (STAT FAX, USA). Transfected cells without DNA and cells without transfection were used as the negative controls.

#### Adenylyl cyclase activity assay

Forty eight hrs after transfection, the cells were exposed to 100 nM vasopressin (Sigma), 2mM isobutylmethylxanthine (Sigma) or 100 µM forskolin (Sigma) for 20 min at 37°C (8, 10). After rinsing with Hanks buffer and lysis by 0.1M HCl, the cAMP was measured using a cAMP (direct) enzyme immunoassay kit (Assay Designs, Ann Arbor, USA). The procedure was performed according to the manufacture's instruction by using a polyclonal antibody against cAMP. After simultaneous incubation at room temperature, the excess reagents were washed away and substrate was added. After a short incubation time the enzyme reaction was stopped and the generated yellow color was read on a microplate reader at 405 nm.

#### Statistical analyses

Statistical comparisons were performed by Student's *t*-test or one-way analysis of variance (ANOVA) followed by Dunnett test. The data are presented as the mean±SEM; p value <0.05 was considered statistically significant.

## RESULTS AND DSCUSSION

**Figure 2:**
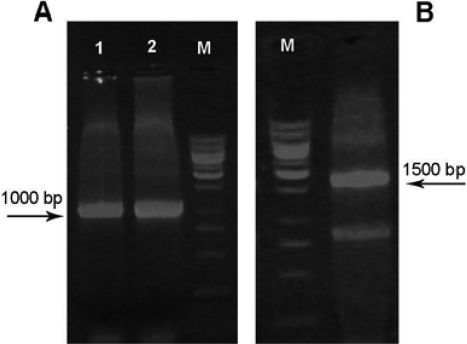
First (a) and nested (b) PCR of DRI mutant at V2 receptor. Products of PCR were electrophoresed on 0.7% agarose gel. In the first PCR by using sense primer of DRI and anti-sense of outer primer in pcDNA3 (step 1) as well as anti-sense primer of DRI and sense of outer primer in pcDNA3 (step 2), a sharp and dense band of 1000 bp was produced. In the nested PCR, by using the first PCR products and sense and anti-sense of inner primer in pcDNA3, a band of 1500 bp was detected. M: molecular weight marker, 250 bp unit.

**Figure 3:**
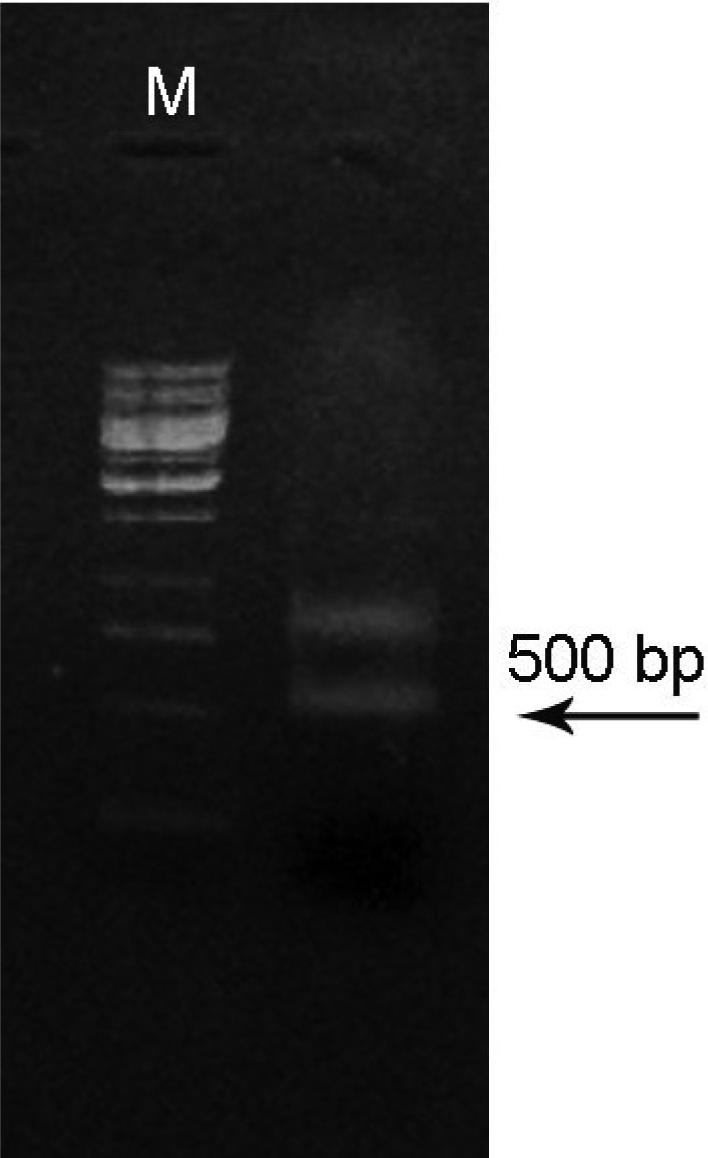
Digestion of the nested PCR of DRI mutant by BamHI. Products of digestion were electrophoresed on 0.7% agarose gel. A band of 500 bp was obtained, indicating the presence of mutation in the PCR product. M: molecular weight marker, 250 bp unit.

The products of the first and second (nested) PCR of DRI mutant are shown in figure 2. Since there was a restriction site for *Bam*H I enzyme at the DRH motif of V2 receptor cDNA*,* the nested PCR product was digested by this enzyme to confirm that the primer design and PCR process are correct (Fig 3).

**Figure 4:**
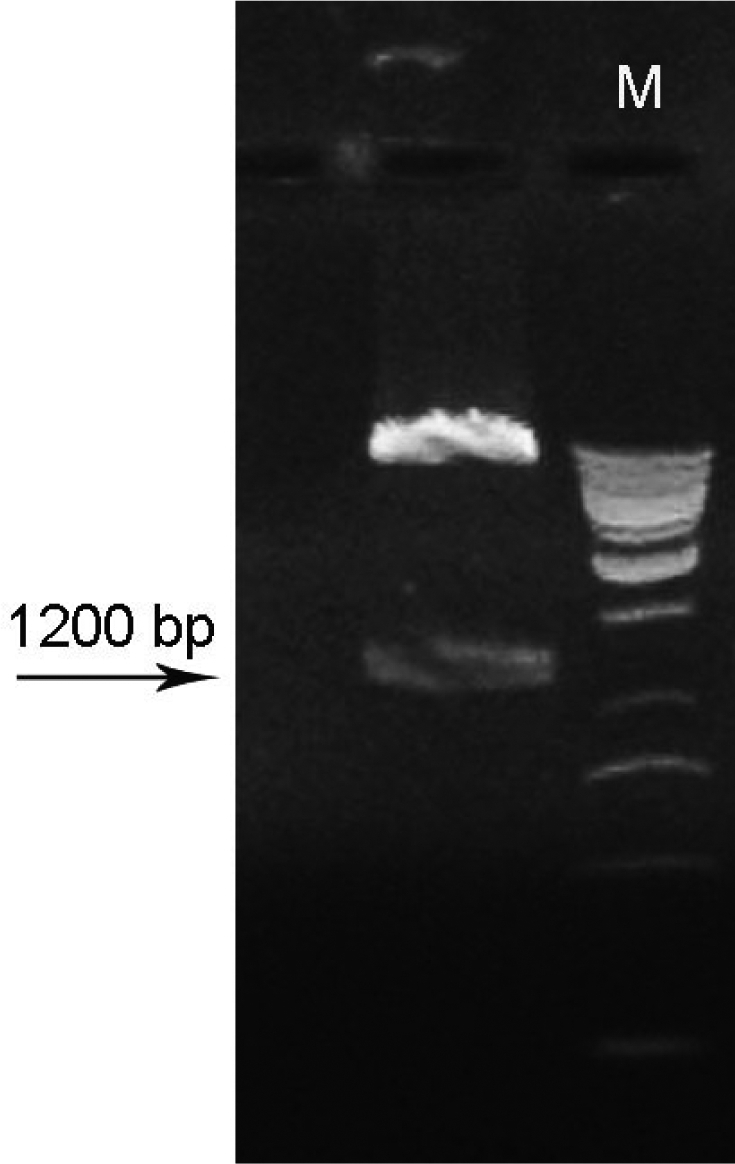
Digestion of pcDNA3 plasmid containing wild type V2R by XbaI-EcoRI enzymes. Products of digestion were electrophoresed on 0.7% agarose gel. The band of V2R (1200 bp) was digested out of the pcDNA3 plasmid. M: molecular weight marker, 250 bp unit.

For vector preparation, pcDNA3 containing the wild type V2R was digested by EcoRI and XbaI enzymes which removed the V2 receptor DNA (1200 bp) out of the pcDNA3 plasmid (5400 bp) (Fig 4).

Following ligation of DRI insert to pcDNA3 vector and transformation to HB101 cells, colonies of bacteria were observed after overnight culture on an agar plate. The clones that were supposed to have uncut pcDNA3 containing DRI mutant were used for plasmid preparation. These plasmids were digested by *Bam*HI enzyme and the presence of a 500 bp band indicated that these plasmids were recombinants. These plasmids were also digested by EcoRI and XbaI enzymes and a 1200 bp band of V2 receptor cDNA was obtained.

**Figure 5:**
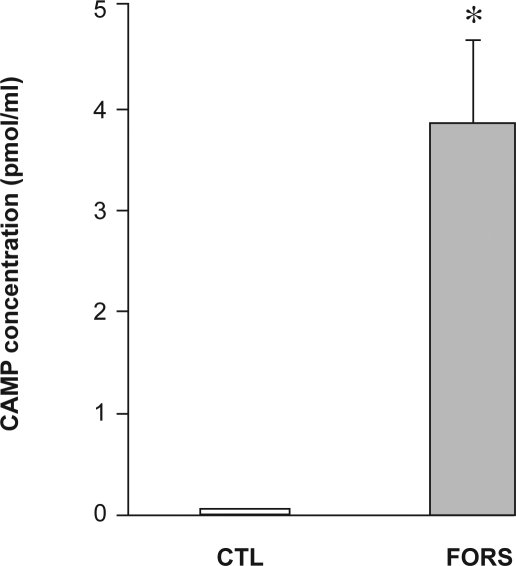
cAMP concentration (pmol/ml) in COS-7 cells. The cells were transfected without plasmid in the absence of forskolin treatment (CTL) or with a plasmid containing wild type cDNA of V2R in the presence of forskolin (FORS) treatment (100 µM) for 20 min at 37°C. Cells were placed at a density of 5×10^5^ cells per well in a 24-well plate and amount of DNA of plasmids was 1000 ng. Data represent the mean±SEM (n=10), *P <0.05.

**Figure 6:**
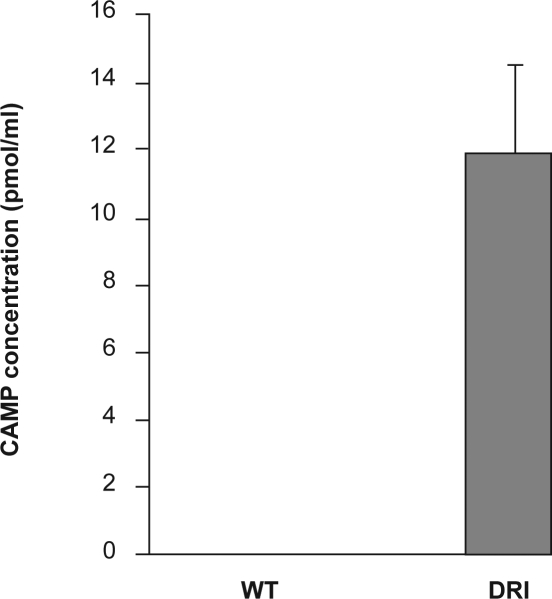
The effects of the mutation of the His residue in the DRH motif of V2 receptor on cAMP concentration (pmol/ml) in COS-7 cells. The cells were transfected with a plasmid containing the wild type cDNA of V2R (WT) or containing DRI mutant cDNA of V2R (DRI). Both groups were treated with methylxanthine (2 mM) and vasopressin (100 nM) for 20 min at 37°C. Cells were placed at a density of 5×10^5^ cells per well in a 24-well plate and DNA amount for each plasmid was 1000 ng. Data represent the mean±SEM (n=20).

The adenylyl cyclase activity assay was performed for functional characterization of DRI mutation on V2R signaling. The standard curve was generated using serial dilutions of standard solutions. The amount of cAMP was inversely related to optical absorbance. The cAMP production measured in COS-7 cells transfected with the wild type and mutant V2R are shown in figures 5 and 6. When compared to the untransfected cells, there was a significant forskolin mediated production of cAMP in cells transfected with the wild type receptor (p<0.05) (Fig 5). On the other hand vasopressin increased the intracellular production of cAMP in cells transfected with the wild type and mutant receptors, no significant difference was observed between these groups (Fig 6).

**Figure 7:**
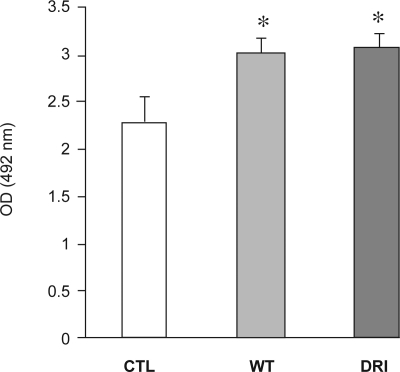
The effects of the mutation of the His residue in the DRH motif on V2 receptor expression in COS-7 cells by ELISA assay. The cells were transfected without any plasmid (CTL) or with a plasmid containing the wild type cDNA of V2R (WT) or containing DRI mutant cDNA of V2R (DRI). Cells were placed at a density of 5 ×10^5^ per well in a 96-well plate and the amount of DNA for each plasmids was 1000 ng. Data represent the optical density (OD) as mean±SEM (n=20), *P <0.05 vs. control.

The results of ELISA for receptor expression assay are shown in figure 7. No significant difference was observed in receptor expression between the wild type and mutant receptors.

DRY motif seems to be an important determinant in the signal transduction of GPCRs (1). However, various site-directed mutagenesis in this region have not revealed a universal role for this motif and its role in GPCR activation and signaling may be different in various receptors (7). To further study the role of this motif in the V2 receptor expression and function, DRI mutant DNA was created in V2R.

In this study, the DRI mutation was cloned into the pcDNA3 vector and functional assays showed no difference in the amount of cAMP production between the wild type and the mutant V2 receptors. For receptor expression, the ELISA method was employed which allows more accurate quantification of receptors at the cell surface in comparison with other techniques (7). The V2 receptor expression also did not change in the presence of this mutation. These results suggest that the role of histidin residue may be not critical in the V2 receptor function.

While different site-directed mutations have been studied in GPCRs, much less is known about the role of the DRH motif in V2R function. The results of V2R studies have shown that mutation of aspartate residue (D136A) in DRH motif increased the cAMP production by 5 folds (11).

Mutations in arginine residue of the V2R may result in decreased expression and loss of function and to produce a constitutively desensitized phenotype (12).

The tyrosine residue has not been extensively studied among the DRY sequence. Some studies have reported that variation of the Tyr residue in the DRY motif may be functionally tolerated (17). However, since the functional relevance of the residues within DRY motif seems to be receptor and context specific (18), further studies are required in each specific receptor to determine the role of individual residue.

In some receptors such as CCR3 chemokine receptor and rat melanin-concentrating hormone receptor 1, tyrosine mutation in DRY motif has resulted in poorly expressed and non-functional receptors (3, 19). Replacements of tyrosine (Y150A) in vasopressin V1a receptor has led to decrease in signal transduction with little effect on ligand binding, signaling, and receptor internalization (7).

Although the functional role of tyrosine residue in DRY motif is unclear, it seems that intramolecular contact of Tyr with Asp and its integrity is important for efficient receptor folding. Moreover although the tyrosine residue does not participate directly in receptor signaling, it may influence maximal responses regarding receptor signaling (7).

In summary, aspartate-arginine-tyrosine triple residues play an important role in the signal transduction pathways of GPCRS, but may not have the same function in all receptors. Regarding V2 receptor it seems that the histidin residue is not critical by itself and its mutation did not affect the V2 receptor function and expression. However further studies are required to look into various mutations of these residues to shed some more light on their exact role in the V2 receptor function.

## References

[1] Birnbaumer M (2001). The V2 vasopressin receptor mutations and fluid homeostasis. Cardiovasc. Res.

[2] Birnbaumer M (1999). Vasopressin receptor mutations and nephrogenic diabetes insipidus. Arch. Med. Res.

[3] Aizaki Y, Maruyama K, Nakano-Tetsuka M, Saito Y (2009). Distinct roles of the DRY motif in rat melanin-concentrating hormone receptor 1 in signaling control. Peptides.

[4] Rovati GE, Capra V, Neubig RR (2007). The highly conserved DRY motif of class A G protein-coupled receptors: beyond the ground state. Mol. Pharmacol.

[5] Bowen-Pidgeon D, Innamorati G, Sadeghi HM, Birnbaumer M (2001). Arrestin effects on internalization of vasopressin receptors. Mol. Pharmacol.

[6] Hawtin SR (2005). Charged residues of the conserved DRY triplet of the vasopressin V1a receptor provide molecular determinants for cell surface delivery and internalization. Mol. Pharmacol.

[7] Birnbaumer M (1995). Mutations and diseases of G protein coupled receptors. J. Recept. Signal Transduct. Res.

[8] Sadeghi HM, Innamorati G, Birnbaumer M (1997). An X-linked NDI mutation reveals a requirement for cell surface V2R expression. Mol. Endocrinol.

[9] Larijani B, Tabatabaei O, Soltani A, Taheri E, Pajouhi M, Bastanhagh MH, Akhondzadeh S, Mahmoodi M, Bandarian F, Mohammadzade N (2005). Comparison of desmopressin (ddavp) tablet and intranasal spray in the treatment of central diabetes insipidus. DARU.

[10] Innamorati G, Sadeghi H, Eberle AN, Birnbaumer M (1997). Phosphorylation of the V2 vasopressin receptor. J. Biol. Chem.

[11] Morin D, Cotte N, Balestre MN, Mouillac B, Manning M, Breton C, Barberis C (1998). The D136A mutation of the V2 vasopressin receptor induces a constitutive activity which permits discrimination between antagonists with partial agonist and inverse agonist activities. FEBS Lett.

[12] Barak LS, Oakley RH, Laporte SA, Caron MG (2001). Constitutive arrestin-mediated desensitization of a human vasopressin receptor mutant associated with nephrogenic diabetes insipidus. Proc. Natl. Acad. Sci. USA.

[13] Oksche A, Dickson J, Schulein R, Seyberth HW, Muller M, Rascher W, Birnbaumer M, Rosenthal W (1994). Two novel mutations in the vasopressin V2 receptor gene in patients with congenital nephrogenic diabetes insipidus. Biochem. Biophys. Res. Commun.

[14] Sadeghi HM, Rabbani M, Jafarian A, Ghafghazi T, Najafzadeh H (2005). Site directed mutagenesis of V2 vasopressin receptor and its cloning using PGEM3Z vector. Biotechnology.

[15] Ausubel FM, Brent R, Kingston RE, Moore DD, Seidman JG, Smith JA, Struhl K (1999). Short protocols in molecular biology.

[16] Sambrook J, Russel DW (2001). Molecular cloning (a laboratory manual).

[17] Ohyama K, Yamano Y, Sano T, Nakagomi Y, Wada M, Inagami T (2002). Role of the conserved DRY motif on G protein activation of rat angiotensin II receptor type 1A. Biochem. Biophys. Res. Commun.

[18] Auger GA, Pease JE, Shen X, Xanthou G, Barker MD (2002). Alanine scanning mutagenesis of CCR3 reveals that the three intracellular loops are essential for functional receptor expression. Eur. J. Immunol.

[19] Römpler H, Yu HT, Arnold A, Orth A, Schöneberg T (2006). Functional consequences of naturally occurring DRY motif variants in the mammalian chemoattractant receptor GPR33. Genomics.

